# A lag bloom pattern of phytoplankton after freshwater input events revealed by daily samples during summer in Qinhuangdao coastal water, China

**DOI:** 10.3389/fmicb.2024.1454948

**Published:** 2024-07-26

**Authors:** Gang Wang, Yike He, Zuoyi Chen, Huixin Liu, Qiuzhen Wang, Chu Peng, Jiabo Zhang

**Affiliations:** ^1^School of Civil Engineering, Tianjin University, Tianjin, China; ^2^The Eighth Geological Brigade, Hebei Geological Prospecting Bureau, Qinhuangdao, China; ^3^Marine Ecological Restoration and Smart Ocean Engineering Research Center of Hebei Province, Qinhuangdao, China; ^4^Ocean College, Hebei Agricultural University, Qinhuangdao, China; ^5^MOE Key Laboratory of Pollution Processes and Environmental Criteria, College of Environmental Science and Engineering, Nankai University, Tianjin, China

**Keywords:** phytoplankton bloom, nutrients, freshwater input, coastal waters, DIP

## Abstract

Phytoplankton blooms have become a global concern due to their negative impacts on public health, aquaculture, tourism, and the economic stability of coastal regions. Therefore, elucidating the shifts in phytoplankton community structure and abundance, as well as their environmental drivers, is crucial. However, existing studies often fail to capture the detailed dynamics of phytoplankton blooms and their environmental triggers due to low temporal observation resolution. In this study, high temporal resolution (daily) samples were collected over 43 days to investigate the influence of environmental factors on phytoplankton in Qinhuangdao in the summer. During the observation period, a total of 45 phytoplankton species were identified, comprising 26 Bacillariophyta species, 16 Dinophyta species, 2 Euglenophyta species, and 1 Chromophyta species. Interestingly, a lag bloom pattern of phytoplankton behind freshwater input was observed across day-to-day samples. Phytoplankton blooms typically lagged 1–3 days behind periods of decreased salinity and nutrient input, suggesting that freshwater influx provides the foundational materials and benefits for these blooms. Moreover, the phytoplankton blooms were triggered by six dominant species, i.e., *Chaetoceros* spp., *Pseudo-nitzschia delicatissima*, *Skeletonema costatum*, *Protoperdinium* spp., *Leptocylindrus minimus*, *Pseudo-nitzschia pungens*, and *Thalassiosira* spp. Consequently, the succession of phytoplankton showed a predominant genera shift in the following sequence: *Nitzschia*, *Protoperdinium*, and *Prorocentrum* – *Skeletonema* – *Pseudo-nitzschia* – *Gymnodinium* – *Leptocylindrus*. Besides that, a deterministic process dominated phytoplankton community assembly across time series, and DIP is a key factor in shifting the phytoplankton community structures in this area. In summary, our study offers high-resolution observations on the succession of phytoplankton communities and sheds light on the complex and differentiated responses of phytoplankton to environmental factors. These findings enhance our understanding of the dynamics of phytoplankton blooms and their environmental drivers, which is essential for the effective management and mitigation of their adverse impacts.

## 1 Introduction

Phytoplanktons are fundamental to ecosystem productivity and the structuring of food webs, and they play a pivotal role in the biogeochemical cycling of key elements such as nitrogen, phosphorus, silicon, and carbon on a global scale (Falkowski et al., 1998; Arrigo, 2005; Hilligsøe et al., 2011). However, the rapid urbanization and economic expansion in coastal regions have markedly transformed phytoplankton communities, promoting the proliferation of specific species that can form extensive blooms (Kroeze et al., 2013). These phytoplankton blooms have significant negative impacts on public health, aquaculture operations, tourism, and the economic stability of coastal areas (Genitsaris et al., 2019). It is reported that phytoplankton blooms are estimated to contribute to an annual global medical cost of $30.3 million (Kouakou and Poder, 2019). Therefore, it is crucial to thoroughly understand phytoplankton ecology in coastal waters to enhance the management and conservation of these environments.

Freshwater inputs have been identified as a pivotal factor in the initiation of phytoplankton blooms (Dai et al., 2023; Zeng et al., 2024). Recent investigations using remote sensing technology have documented a global escalation in the frequency of these blooms (Dai et al., 2023). Analogous patterns have been observed in the coastal regions of China, where both large-scale studies highlighted freshwater influxes, predominantly from rainfall, as the most likely catalyst for phytoplankton proliferation (Zeng et al., 2024). Microcosm experiments further corroborate these findings by demonstrating that freshwater inputs can significantly enhance the growth rate, shift the community composition, and change the size structure of phytoplankton populations (Paczkowska et al., 2020). Despite the compelling evidence highlighting the crucial role of freshwater inputs in inducing phytoplankton blooms, the intricate ecological mechanisms underlying these events remain incompletely understood.

Temporal observations are a powerful tool to identify the environmental drivers behind shifts in phytoplankton abundance and communities (Cui et al., 2018; Nunes et al., 2018; Sin and Yu, 2018; Genitsaris et al., 2019). For example, monthly samples collected in Qinhuangdao have shown that salinity, an indicator of freshwater inputs, plays a particularly significant role in regulating the phytoplankton community (Cui et al., 2018). Additionally, weekly samples from estuaries demonstrate that freshwater inputs can impact phytoplankton abundance and community structure by altering physical factors such as hydrodynamic force, turbidity, and salinity, as well as modifying nutrient supply (Sin and Yu, 2018). In the St. Lucia Estuary, changes in salinity and the availability of nitrogen and phosphorus have been identified as key drivers of shifts in the dominant phytoplankton groups (Nunes et al., 2018). Similarly, in urban Thessaloniki Bay, DIP and NH_4_^+^ levels are positively correlated with phytoplankton blooms (Genitsaris et al., 2019). Our previous daily sampling efforts also indicated that freshwater inputs significantly influence phytoplankton abundance and community composition, with DIP notably affecting the succession of phytoplankton communities and the blooming of dominant species (He et al., 2022). However, the dynamics of phytoplankton communities and abundance are influenced by a complex interplay of factors. Even within the same area, bloom patterns, community structures, and key environmental drivers can vary from year to year. Therefore, sustained daily sampling is essential to capture the detailed dynamics of phytoplankton community shifts and abundance and to accurately identify their ecological drivers.

Qinhuangdao, located along the Bohai Sea and renowned as a seaside tourist destination in northern China, experiences relatively weak water exchange (Tao, 2006). The region’s coastline is fed by over 17 rivers, which contribute substantial nutrient loads to the coastal waters. Over the past decade, the area has frequently experienced algal blooms, significantly affecting local economic development. These blooms typically begin in April and peak in July (Cui et al., 2018). A particularly notable event was the large-scale brown tide from June to August 2012, which affected 3,400 km^2^ and lasted for 73 days (Zhang et al., 2013). In recent years, smaller algal blooms have also been observed more frequently along the Qinhuangdao coast (Department of Natural Resources of Heibei Province (Ocean Administration), 2023). Thus, Qinhuangdao serves as an excellent region for investigating the ecology of phytoplankton and the dynamics of algal blooms.

In this study, high-resolution time series samples (collected daily) were obtained during the summer, the season with the highest incidence of algal blooms, from a coastal area in Qinhuangdao, China, influenced by a small river. Phytoplankton were identified and counted at the species level using morphological examinations and microscopy. Simultaneously, various environmental factors, including pH, temperature, salinity, and nutrient concentrations, were monitored. The objectives of this study were as follows: (1) to capture the bloom pattern of phytoplankton in the Qinhuangdao coastal area, (2) to reveal the major factors that triggered the blooms and community successions of the phytoplankton, and (3) to provide important insights for developing management strategies to control phytoplankton blooms in coastal waters.

## 2 Materials and methods

### 2.1 Study area and water sampling

**Figure 1:**
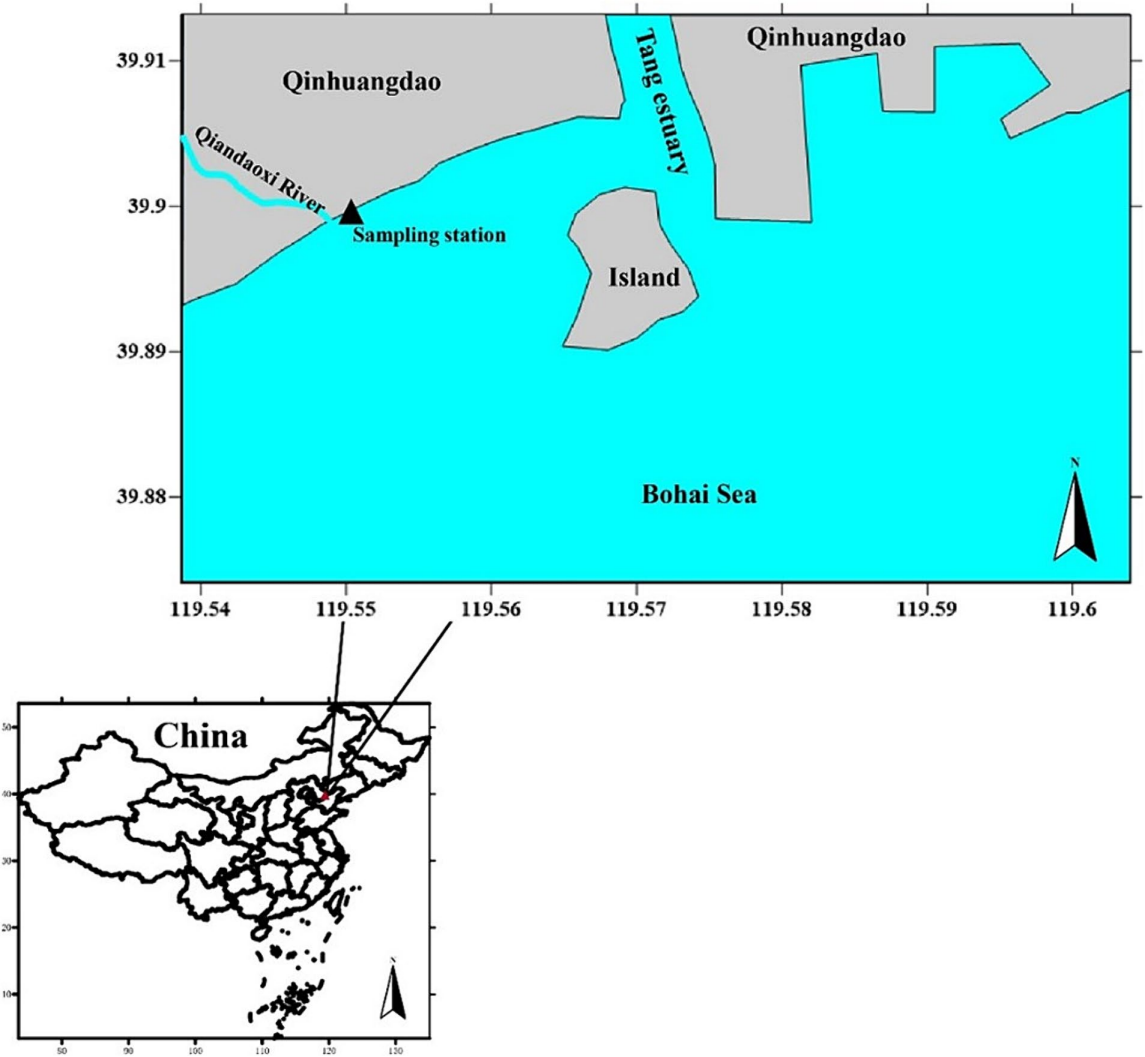
Map of the sampling station (He et al., 2022).

The sampling site was located in Jinmeng Bay, Qinhuangdao, Hebei Province, and was influenced by the Qiandao River. The island located in the Tang River estuary is an artificial island (Figure 1). Water samples were systematically collected daily from a fixed location between 10 July 2021 and 23 August 2021. Approximately 2.5 L of near-surface seawater (at a depth of 1 m) were gathered each day at 3:00 p.m. All collected samples were immediately placed in a cooler with ice packs, maintaining a temperature of approximately 4°C, and were transported to the laboratory within 2 h for subsequent analysis.

### 2.2 Environmental parameters

Salinity, water temperature, and pH were measured on-site using a portable multi-parameter water quality monitoring instrument (YSI Pro Series, USA). For nutrient analysis, approximately 500 mL of seawater was filtered through 0.45 μm pore-size membranes. The concentrations of silicate (Si), nitrite (NO₂^−^), nitrate (NO₃^−^), and ammonium (NH₄^+^) were quantified using a QuAAtro nutrient autoanalyzer (Seal Analytical Ltd., Germany), following the methods outlined by He et al. (2019). The combined concentrations of NO₂^−^, NO₃^−^, and NH₄^+^ were utilized to compute the dissolved inorganic nitrogen (DIN) levels. Dissolved inorganic phosphorus (DIP) was monitored in accordance with the “Specification for Oceanographic Survey” (GB/T 12763.4–2007). Rainfall data were sourced from the Chinese Weather Bureau.^1^

### 2.3 Phytoplankton identification

Phytoplankton species were taxonomically identified based on morphological examinations, adhering to the guidelines stipulated in the “Technical Specification for Red Tide Monitoring in China.” Following collection, phytoplankton samples were preserved in 1.5% Lugol’s solution for a minimum of 24 h to facilitate sedimentation of phytoplankton cells. Subsequently, approximately 10 mL of concentrated phytoplankton samples were withdrawn from the bottom layer. Taxonomic determinations and enumeration (cells·L^−1^) were conducted using a microscope (OLYMPUS CX31, Japan), following the established methodology outlined by Utermöhl (Utermöhl, 1958). Species identification was pursued to the extent possible, with all specimens identified to at least the genus level.

### 2.4 Data analysis

The phytoplankton species diversity indices of Shannon–Wiener and richness were calculated by *vegan* package using RStudio (R Development Core Team, 2011). The dominance index (*Y*) was calculated according to Eq. (1). Non-metric multidimensional scaling (NMDS) was performed by a *vegan* package using RStudio (R Development Core Team, 2011) to visualize the phytoplankton communities in different groups. Adonis analyses and permutation MANOVAs were applied to test the distinction of phytoplankton in different groups. A mental test was used to analyze the correlation between phytoplankton community structure and environmental factors. Cross-correlation analysis was used to reveal the lag bloom pattern of phytoplankton after freshwater input events using RStudio (R Development Core Team, 2011). The Spearman test was used to analyze the correlation between different environmental factors and dominant species. The taxonomic normalized stochasticity ratio (tNST) was calculated based on the Bray–Curtis distance using the NST package (Ning et al., 2019) in R. The tNST value was used to estimate the ecological stochasticity, with 50% as the cutoff between more deterministic (<50%) and more stochastic (>50%) assemblies.


(1) $$Y=\frac{n_{i}}{N}\times f_{i}$$

where *N* represents the number of total species detected during our observation time, *n_i_* represents the abundance of species *i*, and *f_i_* represents the detectable rate of species *i*.

## 3 Results

### 3.1 Environmental factors

**Figure 2:**
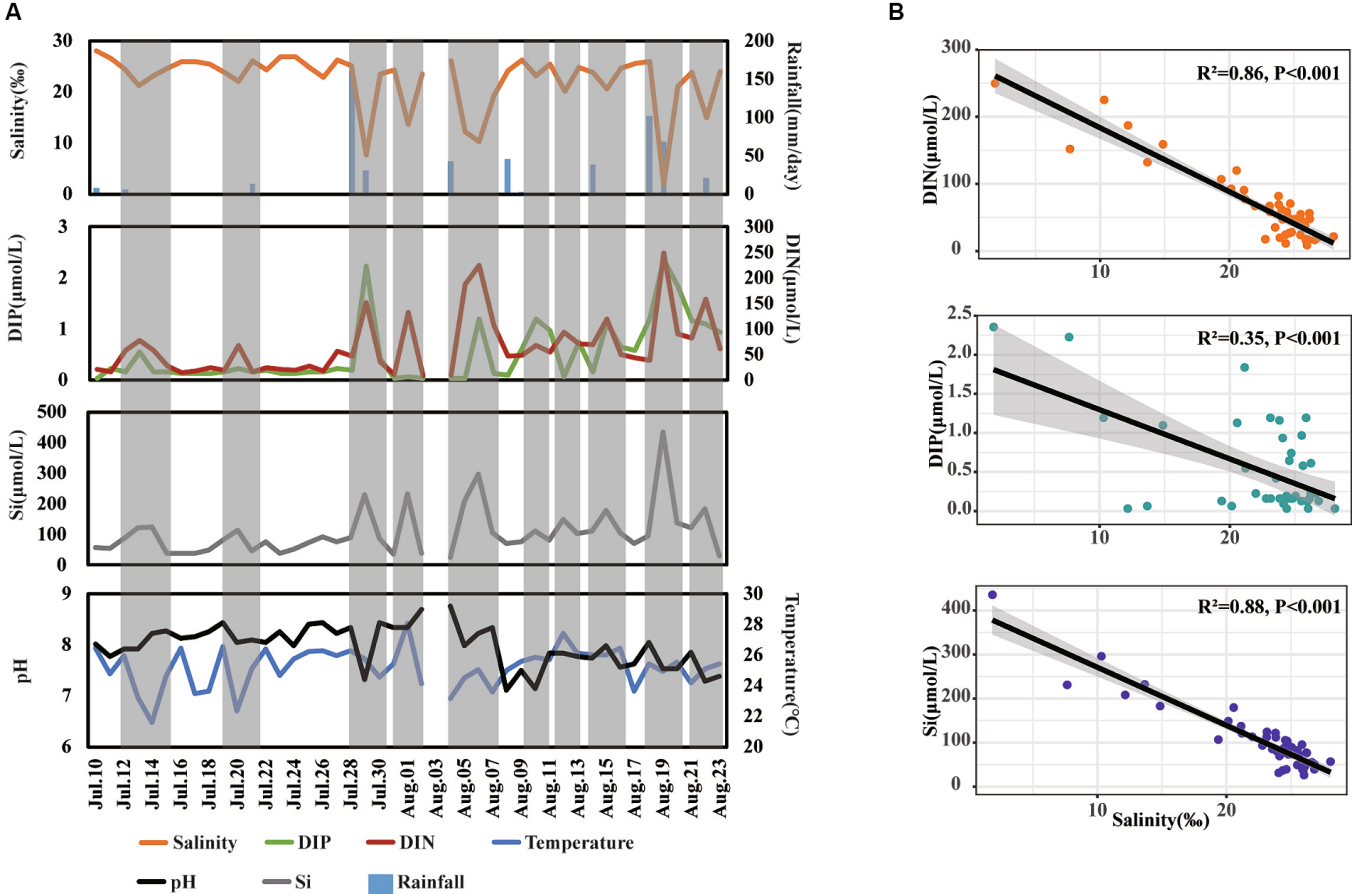
Shift **(A)** and relationship **(B)** of different environmental factors across day-to-day time. The gray shadow in panel **(A)** represents special periods when salinity decreased.

The variations in environmental factors over the day-to-day time series were first analyzed (Figure 2A). The concentrations of DIN, DIP, and Si ranged from 8.50 to 249.79 μmol/L, 0.03 to 2.35 μmol/L, and 26.39 to 435.71 μmol/L, respectively. For physical parameters, salinity, temperature, and pH ranged from 1.87 to 26.82‰, 23.70 to 29.20°C, and 6.48 to 8.42, respectively (Figure 2A). Additionally, there were 17 days of rainfall, with daily precipitation ranging from 0.1 to 160.6 mm.

During our observation period, we recorded 10 instances of decreased salinity, indicating freshwater inputs in this area. Notably, during these periods of decreased salinity, the concentrations of DIP, DIN, and Si increased several folds compared to normal days. Furthermore, the concentrations of DIN and Si showed a strong negative correlation with salinity levels (*R*^2^ > 0.85, *p* < 0.001), while the concentrations of DIP showed a relatively weaker negative correlation with salinity levels (*R*^2^ = 0.35, *p* < 0.001) (Figure 2B) These findings suggest that freshwater inputs significantly enhance nutrient levels, with a more pronounced influence on the concentrations of DIN and Si than on DIP in coastal waters.

### 3.2 Abundance of phytoplankton

**Figure 3:**
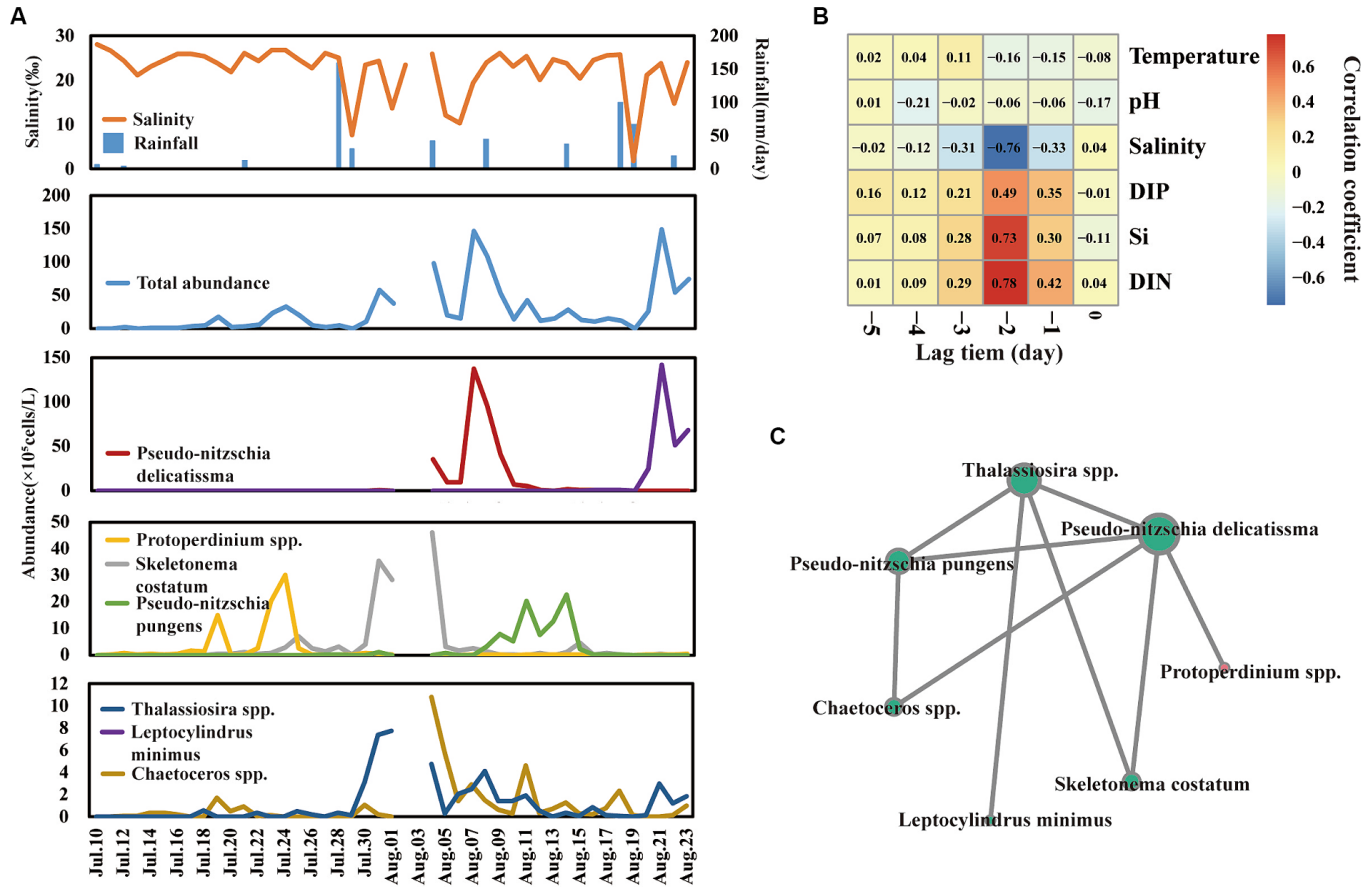
Shift of total phytoplankton and dominant species abundance across time series **(A)**. Cross-correlation analysis (CCA) reveals the lag bloom pattern of total phytoplankton abundance **(B)**. Network reveals the co-relationship among dominant species **(C)**.

The total abundance of phytoplankton ranged from 2.0 × 10^4^ to 1.49 × 10^7^ cells/L (Figure 3A), which was comparable to our previous study in this field (He et al., 2022). Notably, there were nine phytoplankton bloom events that happened on the 19th, 24th, and 31st of July and the 5th, 7th, 11th, 14th, 21st, and 23rd of August, respectively. Notably, the bloom event was not triggered by the same species. According to the analysis of dominant species (*Y* > 0.02) abundance (Figure 3A), nine bloom events were triggered by seven dominant species, including *Chaetoceros* spp., *Pseudo-nitzschia delicatissima*, *Skeletonema costatum*, *Protoperdinium* spp., *Leptocylindrus minimus*, *Pseudo-nitzschia pungens*, and *Thalassiosira* spp. For example, *Protoperdinium* spp. dominated the bloom events on 19 July and 24 July. *Skeletonema costatum* and *Thalassiosira* spp. co-dominated bloom event on 31 July. Furthermore, network analysis (Figure 3C) of those seven species showed that *Pseudo-nitzschia delicatissima*, leading to a big bloom event on 7 August, significantly positively correlated with other dominant species except *Leptocylindrus minimus*.

Interestingly, a lag bloom pattern of phytoplankton behind freshwater input was observed across day-to-day time samples. Phytoplankton blooms usually lag behind the time of salinity decrease and nutrient inputs. For example, the salinity value dramatically decreased to 1.87 on 19 August, and the abundance of phytoplankton bloomed to 1.49 × 10^7^ cells/L after 2 days. Furthermore, cross-correlation analysis, which could analyze the correlation between a time series and a lagged version of another time series, was used to analyze the lag bloom pattern (Figure 3B). The level of salinity, DIN, Si, and DIP showed a high correlation coefficient with phytoplankton abundance in lag 2 days, suggesting that there is a high likelihood of phytoplankton blooms occurring approximately 3 days after freshwater inputs.

Notably, we also found four potential toxic species (Supplementary Figure S1), including *Prorocentrum minimum*, *Gymnodinium catenatum*, *Chattonella marina,* and *Akashiwo sanguinea*. Of those four species, *Gymnodinium catenatum*, *Chattonella marina,* and *Akashiwo sanguinea* were occasionally detected and showed a relatively low abundance (<1.00 × 10^4^ cells/L). Nevertheless, *Prorocentrum minimum* was detected in 16 samples, and the peak abundance was 8.64 × 10^5^ cells/L, which was near to the threshold value (1.00 × 10^6^ cells/L) of red tide according to “Technical specification for red tide monitoring in China.”

### 3.3 Diversity of phytoplankton

**Figure 4:**
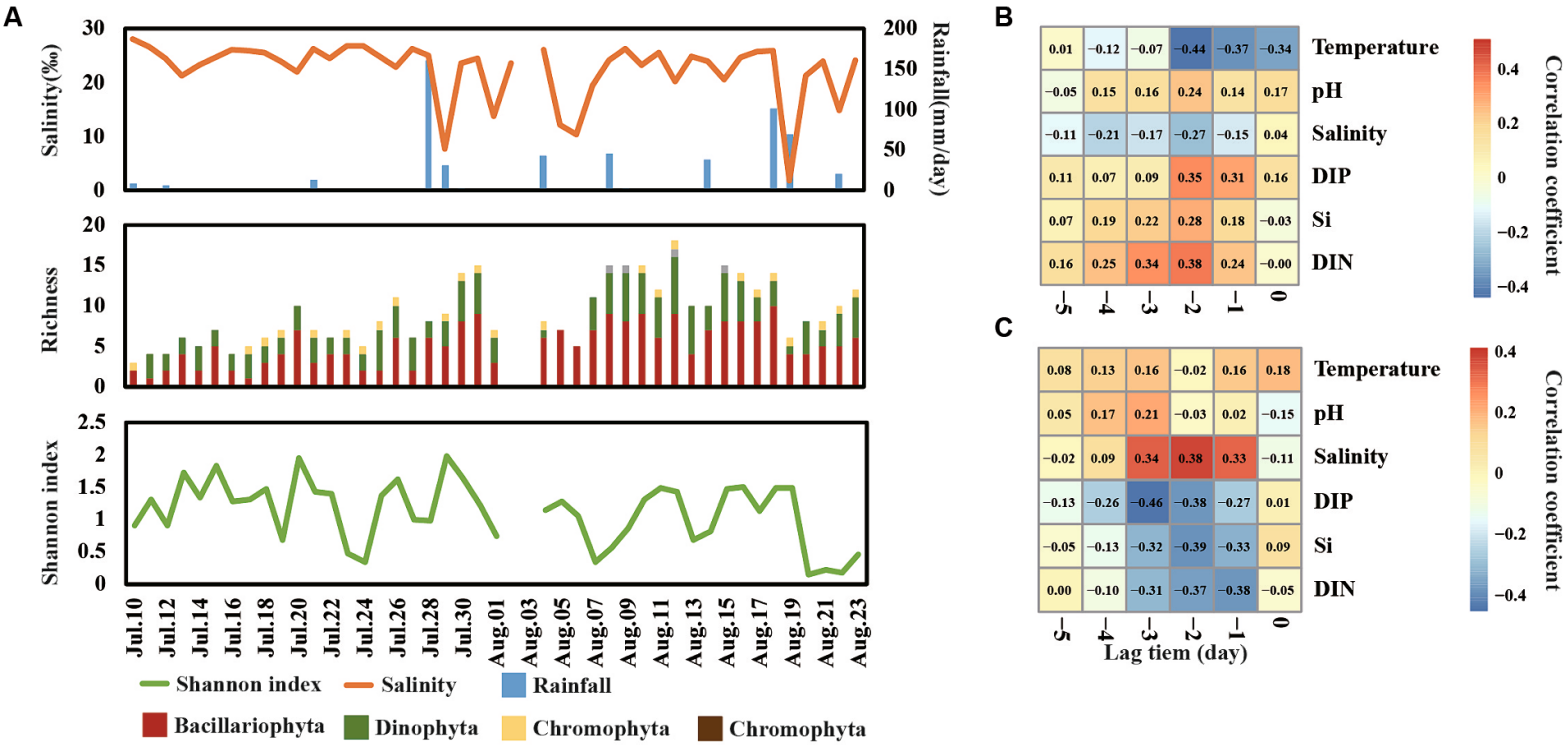
Shift of richness and Shannon index of phytoplankton communities across day-to-day time **(A)**. Cross-correlation analysis (CCA) reveals the lag change pattern of phytoplankton richness **(B)** and Shannon index **(C)**.

A total of 45 species were detected in Jinmeng Bay during our observation period and 26, 16, 2, and 1 species were divided into Bacillariophyta, Dinophyta, Chromophyta, and Euglenophyta, respectively (Figure 4A). Moreover, Bacillariophyta was detected in all samples, and the number of species belonging to Bacillariophyta ranged from 1 to 10 across time series. Dinophyta was detected in most samples and the number of species belonging to it ranged from 0 to 7 across time series. There is one species belonging to Euglenophyta that was detected in more than half samples. Nevertheless, two Chromophyta species were only detected in three samples.

The Shannon index varied from 0.15 to 2.00 in our observation period (Figure 4A). It was notable that the Shannon index showed a significantly negative relationship with the total abundance of phytoplankton. For example, the total abundance of phytoplankton reached a peak on 7 August, while the Shannon index was at a low value on this day. This finding is consistent with our previous studies in this area (He et al., 2022), suggesting that minority species bloom occupied the niche of other phytoplankton and decreased the diversity. Moreover, the level of salinity, DIN, Si, and DIP showed the strongest correlation with richness and Shannon diversity in lag 2–3 days (Figures 4BC), suggesting that most change of diversity would happen in 2 days after the change of salinity, DIN, Si, and DIP.

### 3.4 Phytoplankton community

**Figure 5:**
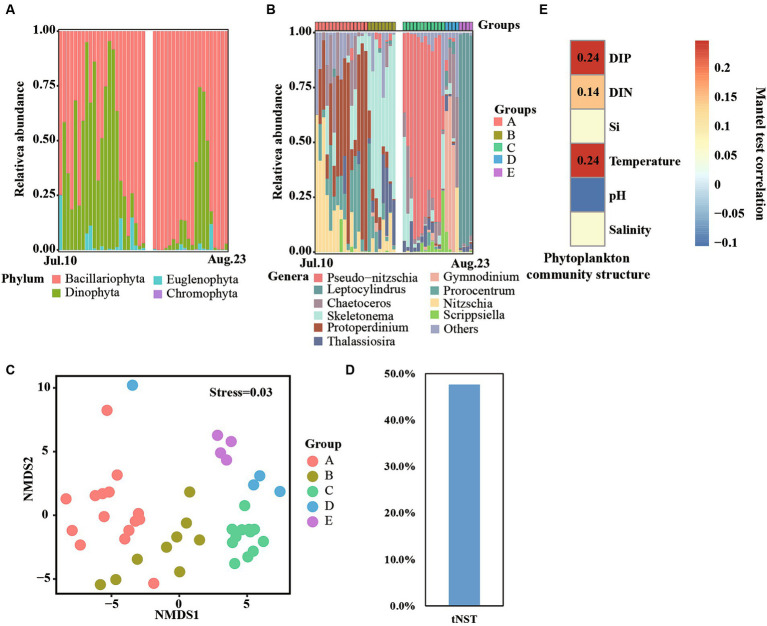
Phytoplankton communities at phylum **(A)** and genera **(B)** levels across time series. Non-metric multidimensional scaling (NMDS) reveals different phytoplankton communities **(C)**. Null model analyses reveal the taxonomic normalized stochasticity ratio (tNST) of phytoplankton community assemblages, i.e., the relative importance of stochastic processes in the community assembly across day-to-day samples **(D)**. Mantel test reveals the correlation between phytoplankton community structure and environmental factors **(E)**.

Bacillariophyta and Dinophyta were the dominant phyla in our observation period (Figure 5A). Bacillariophyta was the most persistent phylum and was detected in all samples, accounting for more than 85% of the total abundance. As well, Bacillariophyta contributed more than 50% phytoplankton abundance in approximately 70% of samples. Notably, Dinophyta were also detected in most samples and bloomed on some days. For details, Dinophyta was detected in more than 90% of samples and bloomed on 11–25 July and 15–19 August, respectively. Moreover, 12 samples showed more than 50% relative abundance.

At the genera level, a clear succession progress of the phytoplankton community was observed and the communities were divided into A, B, C, D, and E groups according to the succession of the dominant genera across time series (Figure 5B). For details, samples collected from 10 to 24 July were divided into group A, and *Nitzschia*, *Protoperdinium,* and *Prorocentrum* were the dominant genera of this group. Group B contained samples collected from 25 July—1 August, and *Skeletonema* was identified as the most abundant genera. Moreover, *Protoperdinium*, *Prorocentrum*, and *Thalassiosira* were also detected in some samples. Samples collected on 4 to 15 August comprised group C, which was dominated by *Pseudo-nitzschia*. Samples collected on 16–19 August and 20–23 August belonged to groups D and E, respectively. Meanwhile, *Gymnodinium* and *Leptocylindrus* dominated the phytoplankton community structure in those two groups, respectively. NMDS analyses also found that all samples were clustered into five groups (Figure 5C), and significant (*p* < 0.01) differences were observed between them (Supplementary Table S2), which consisted of the results of phytoplankton community structure at the genera level.

The value of tNST, representing the relative importance of stochastic processes in governing taxonomic community structure, was 47% (Figure 5D), lower than 50%, suggesting deterministic selection in governing the taxonomic community structure. Furthermore, significant correlations (*p* < 0.01, Mantal test) were observed among phytoplankton community structures and DIN, DIP, and temperature (Figure 5E), indicating that these environmental factors influenced the succession of phytoplankton community structures.

## 4 Discussion

### 4.1 Lag bloom pattern of phytoplankton behind freshwater input

Phytoplankton blooms have been considered one of the most prevalent ecological problems in coastal regions. In recent years, research has observed an increased trend of phytoplankton bloom frequency on a global scale (Kahru and Mitchell, 2008; Dai et al., 2023). However, the mechanism of this phenomenon still cannot be fully understood. A possible reason is that it is hard to capture environmental factors before phytoplankton bloom, which was considered to play a key role in explaining the phytoplankton blooms. In this study, high-resolution time series samples presented complete data about the progress of phytoplankton blooms, which helps people better understand the mechanism of phytoplankton blooms in coastal regions.

Several studies have demonstrated that phytoplankton blooms are correlated with freshwater inputs and nutrients taken by them. For example, recent research based on remote sensing demonstrated that phytoplankton bloom on a global scale is significantly associated with rainfall events, which led to the high input of freshwater providing abundant nutrients in coastal waters (Dai et al., 2023). However, research has not yet confirmed how long phytoplankton will bloom after freshwater inputs. In this study, we repeatedly observed the association between freshwater inputs and phytoplankton blooms. Furthermore, phytoplankton bloom usually happens in 1–3 days after freshwater inputs in coastal water. The explanation for this phenomenon can be attributed to three reasons. First, freshwater inputs can dilute the phytoplankton, which has been identified as one important factor in regulating phytoplankton abundance in the estuary region. Moreover, this study also found that low levels of phytoplankton abundance and salinity usually cooccurred (Figure 3A). Second, phytoplankton bloom fellows Limiting Factor Principle. Its bloom is a comprehensive progress, which depended on a number of factors, including nutrients (Carstensen et al., 2015), sunlight (Fang et al., 2008), salinity (Gasiūnaitė et al., 2005), water temperature (Trombetta et al., 2019) and so on. When nutrients are the only limiting factor, nutrient inputs can trigger phytoplankton blooms. Otherwise, it still needs to satisfy other factors to induce phytoplankton blooms. Third, even if all environmental factors satisfied the phytoplankton bloom conditions, it still takes several hours for the proliferation of phytoplankton cells. For example, according to the laboratory experiment, the growth rates of *Skeletonema costatum* ranged from 0.48 to 1.50 day^−1^, suggesting that the abundance of *Skeletonema costatum* enhanced one order needs at least 1.5 days (Vlas et al., 2020). Overall, our research observed a lag bloom pattern of phytoplankton and partly explained this phenomenon. However, due to the lack of hydrodynamic force, e.g., inflows and tide data, and weather data, this study mainly focused on the relationship between nutrients and phytoplankton bloom. In future, comprehensive data were needed to better explore the mechanism of a phytoplankton bloom.

### 4.2 Deterministic process of dominant phytoplankton community assembly

Freshwater inputs were the most significant disturbance during our observation period. These disturbances significantly decreased salinity levels but increased the levels of nutrients such as DIN, DIP, and silica (Si). Additionally, the nutrient structure in the area was altered. This finding is consistent with several studies on inflows impacting coastal waters (Miller et al., 2008; Hemraj et al., 2017). Notably, changing those environmental factors played an important role in shaping the phytoplankton community structure (Wang et al., 2021). Consistently, our research also found that deterministic process dominant phytoplankton community assembly (Figure 5D) and a number of nutrients showed significant correlation with phytoplankton community structure (Figure 5E).

DIP plays a key role in shaping the phytoplankton community. In this research area, the N/P ratio ranged from 32 to 5,803 (Supplementary Table S1), which is extremely higher than the Redfield ratio (Redfield, 1958), suggesting that DIP is the limiting environmental factor in this region. Moreover, DIP showed the strongest correlation with phytoplankton community structure (Figure 5D), consistent with our previous study based on day-to-day samples in 2021 (He et al., 2022). Therefore, DIP is a key factor in regulating the shift of phytoplankton blooms and community structure.

We also observed that a similar structure of phytoplankton species dominated the bloom. For details, nine bloom events were triggered by seven dominant species, namely, *Chaetoceros* spp., *Pseudo-nitzschia delicatissma*, *Skeletonema costatum*, *Protoperdinium* spp., *Leptocylindrus minimus*, *Pseudo-nitzschia pungens*, and *Thalassiosira* spp. Notably, except for one Dinophyta species, *Protoperdinium* spp., the other six bloomed Bacillariophyta species shared similar structures, i.e., the small size of a single cell but linking to long chains, suggesting that those specific structures are advantageous in interspecific competition. Small single cell size can help phytoplankton rapidly adsorb nutrients in an impulse nutrient inputs environment, which increases their competitive ability in this environment (Litchman and Klausmeier, 2008). Furthermore, chain structure also has an advantage against predation; data on copepods and zooplankton in Narragansett Bay show that increases in size occur right after increases in zooplankton concentration (Sonnet et al., 2022). Chain structure can regulate the buoyancy of phytoplankton and help them get more sunlight in coastal waters (Padisák et al., 2003). Therefore, phytoplankton with small single-cell and chain structures can be more adapted to estuary environments with impulse nutrient inputs.

### 4.3 The potential risk of harmful algal blooms

In this study, we also found 4 toxic species, i.e., *Prorocentrum minimum*, *Gymnodinium catenatum*, *Chattonella marina,* and *Akashiwo sanguinea* (Oshima et al., 1993; Kahn et al., 1995; Heil et al., 2005; Tang and Gobler, 2015). Particularly, *Prorocentrum minimum* was detected in 16 days, and the peak abundance was 8.64 × 10^5^ cells/L, which was near to the threshold value (1.00 × 10^6^ cells/L) of red tide according to “Technical specification for red tide monitoring in China.” *Prorocentrum minimum* has been observed to bloom worldwide. Even its abundance was not higher than the thread value in this study, considering that *Prorocentrum minimum* was a toxin species, the toxin produced by it can be accumulated in scallops by the food chain, which may further influence public health and people still need to be concerned of Qinhuangdao coastal waters.

## 5 Conclusion

In this study, daily samples were collected to investigate the phytoplankton bloom pattern and succession of phytoplankton communities. During the observation period, a total of 45 phytoplankton species were identified, comprising 26 Bacillariophyta species, 16 Dinophyta species, 2 Euglenophyta species, and 1 Chromophyta species. Interestingly, a lag bloom pattern of phytoplankton behind freshwater input was observed across day-to-day samples. Phytoplankton blooms typically lagged 1–3 days behind periods of decreased salinity and nutrient input. Moreover, the phytoplankton blooms were triggered by six dominant species, i.e., *Chaetoceros* spp., *Pseudo-nitzschia delicatissima*, *Skeletonema costatum*, *Protoperdinium* spp., *Leptocylindrus minimus*, *Pseudo-nitzschia pungens*, and *Thalassiosira* spp. Consequently, the succession of phytoplankton showed a predominant genera shift in the following sequence: *Nitzschia*, *Protoperdinium*, and *Prorocentrum* – *Skeletonema* – *Pseudo-nitzschia* – *Gymnodinium* – *Leptocylindrus*. Notably, deterministic process dominated phytoplankton community assembly across time series and DIP is a key factor to shift the phytoplankton community structures in this area. Overall, our results provide high-resolution observation about the succession of phytoplankton communities and shed some light on the complex and partitioning responses of phytoplankton to environmental factors.

## Data availability statement

The original contributions presented in the study are included in the article/Supplementary material, further inquiries can be directed to the corresponding authors.

## Author contributions

GW: Writing – original draft. YH: Conceptualization, Investigation, Supervision, Visualization, Writing – review & editing. ZC: Data curation, Methodology, Project administration, Writing – original draft. HL: Formal analysis, Project administration, Software, Writing – review & editing. QW: Data curation, Investigation, Validation, Visualization, Writing – original draft. CP: Data curation, Methodology, Visualization, Writing – original draft. JZ: Conceptualization, Funding acquisition, Resources, Writing – review & editing.

## Footnotes

^1^ http://www.weather.com.cn
