# Corrigendum: A lag bloom pattern of phytoplankton after freshwater input events revealed by daily samples during summer in Qinhuangdao coastal water, China

**DOI:** 10.3389/fmicb.2025.1601738

**Published:** 2025-04-22

**Authors:** Gang Wang, Yike He, Zuoyi Chen, Huixin Liu, Qiuzhen Wang, Chu Peng, Jiabo Zhang

**Affiliations:** ^1^School of Civil Engineering, Tianjin University, Tianjin, China; ^2^The Eighth Geological Brigade, Hebei Geological Prospecting Bureau, Qinhuangdao, China; ^3^Marine Ecological Restoration and Smart Ocean Engineering Research Center of Hebei Province, Qinhuangdao, China; ^4^Ocean College, Hebei Agricultural University, Qinhuangdao, China; ^5^MOE Key Laboratory of Pollution Processes and Environmental Criteria, College of Environmental Science and Engineering, Nankai University, Tianjin, China

**Keywords:** phytoplankton bloom, nutrients, freshwater input, coastal waters, DIP

In the published article, there was an error in the Funding statement: “This research was funded by the key R&D Program of Hebei Province, China (Nos. 237301D and 2137302D)”. The correct Funding statement appears below.

