# Supplementary material for: A lag bloom pattern of phytoplankton after freshwater input events revealed by daily samples during summer in Qinhuangdao coastal water, China
Source: Front Microbiol. 2024 Jul 26;15:1454948. doi: 10.3389/fmicb.2024.1454948 (PMC11310044; doi:10.3389/fmicb.2024.1454948)
Supplement: Supplementary file 1 [file Data_Sheet_1.docx]

Supplementary Material

**A lag bloom pattern of phytoplankton after freshwater input events revealed by daily samples during summer in Qinhuangdao coastal water, China**

Gang Wang^1,2,3^, Yike He^2,3,*^, Zuoyi Chen^2,3^, Huixin Liu^2,3^, Qiuzhen Wang^4^, Chu Peng^5^, Jiabo Zhang^1,2,*^

^1^School of Civil Engineering, Tianjin University, Tianjin 300072, China

^2^The Eighth Geological Brigade, Hebei Geological Prospecting Bureau, Qinhuangdao 066001, China

^3^Marine Ecological Restoration and Smart Ocean Engineering Research Center of Hebei Province, Qinhuangdao 066001, China

^4^Ocean College, Hebei Agricultural University, Qinhuangdao 066001, China

^5^MOE Key Laboratory of Pollution Processes and Environmental Criteria, College of Environmental Science and Engineering, Nankai University, Tianjin 300350, China

**Key words**: phytoplankton bloom, nutrients, freshwater input, coastal waters, DIP

^*^Corresponding author:

E-mail: yikehe@foxmail.com

longes@163.com


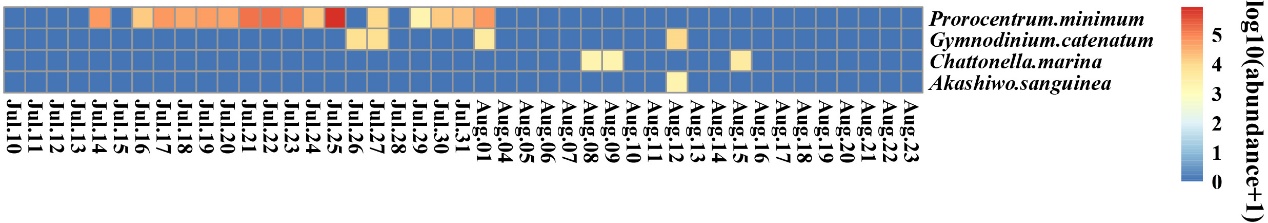


Figure S1 Abundance of toxic species across time series.

Table S1 The environmental factors in coastal water

|  | Minimum value | Maximum value | Average value |
| --- | --- | --- | --- |
| Temperature(℃) | 23.70 | 29.20 | 26.59 |
| pH | 6.48 | 8.42 | 7.56 |
| Salinity(‰) | 1.87 | 26.82 | 22.38 |
| DIP(μmol/L) | 0.03 | 2.35 | 0.52 |
| NO_3_(μmol/L) | 4.93 | 166.43 | 39.89 |
| NH_4_(μmol/L) | 3.00 | 94.29 | 24.13 |
| NO_2_(μmol/L) | 0.07 | 8.79 | 2.03 |
| Si(μmol/L) | 26.39 | 435.71 | 107.94 |
| DIN(μmol/L) | 8.93 | 249.79 | 66.05 |
| N/P | 32.20 | 5803.64 | 395.28 |

Table S2 Pairwise comparisons for phytoplankton community using permutation MANOVAs

|  | A | B | C | D |
| --- | --- | --- | --- | --- |
| B | 0.0025 | - | - | - |
| C | 0.0025 | 0.0025 | - | - |
| D | 0.0029 | 0.0044 | 0.0029 | - |
| E | 0.0025 | 0.0037 | 0.0029 | 0.016 |
